# The complete mitochondrial genome of *Aegialites californicus* (Motchoulsky, 1845) (insecta: coleoptera: salpingidae)

**DOI:** 10.1080/23802359.2024.2309255

**Published:** 2024-01-30

**Authors:** Marianne Nilsen Haugen, Vladimir I. Gusarov, Torsten Hugo Struck

**Affiliations:** Natural History Museum, University of Oslo, Oslo, Norway

**Keywords:** Complete mitochondrial genome, Coleoptera, *Aegialites*

## Abstract

The flightless intertidal beetle genus *Aegialites* (family Salpingidae) is distributed along the Northern Pacific coasts, from California to Alaska and from Northern Japan to Kamchatka. Systematics of *Aegialites* and its phylogenetic relationships to other members of Salpingidae are unclear, and little genetic information is available. We here present the first complete mitochondrial genome of this genus, represented by *Aegialites californicus* (Motchoulsky, 1845) from Sonoma County, California, U.S.A. The complete mitochondrial genome of *A. californicus* is 15,899 bp long and comprises 13 protein-coding (PCG), two ribosomal RNA (rRNA) and 22 transfer RNA (tRNA) genes. The phylogenetic analysis places *A.californicus* as sister to other members of family Salpingidae. The mitochondrial genome sequence of *A. californicus* will contribute to future phylogenetic and taxonomic studies of genus *Aegialites*, family Salpingidae and superfamily Tenebrionoidea.

## Introduction

The beetle genus *Aegialites* Mannerheim, 1853 (Salpingidae) comprises flightless beetles, which inhabit rocks and rock crevices in the supralittoral zone of rocky shores. They are recorded from a wide range of Northern Pacific coasts, from California to Alaska and from Northern Japan to Kamchatka (Zerche 2004). The supralittoral zone on rocky shores is a challenging environment with daily fluctuations in temperature, humidity and salinity as well as exposure to wave actions of different strength. The *Aegialites* beetles have therefore required special morphological adaptations like widely separated coxae, long tarsi and large claws.

Until recently, only four species have been recognized in the genus *Aegialites* (Spilman 1967). Zerche (2004) conducted a revision of the genus based on morphology and increased the total number of species from four to thirty. Zerche (2004) changed the previously accepted paradigm of few widespread and variable species in *Aegialites* to that of many locally distributed species. The validity of Zerche’s hypothesis has never been tested using molecular data. The only work that used molecular data in *Aegialites* (Hojito et al. 2010) compared different populations from Hokkaido using one mitochondrial gene (*nad2*).

**Figure 1.**
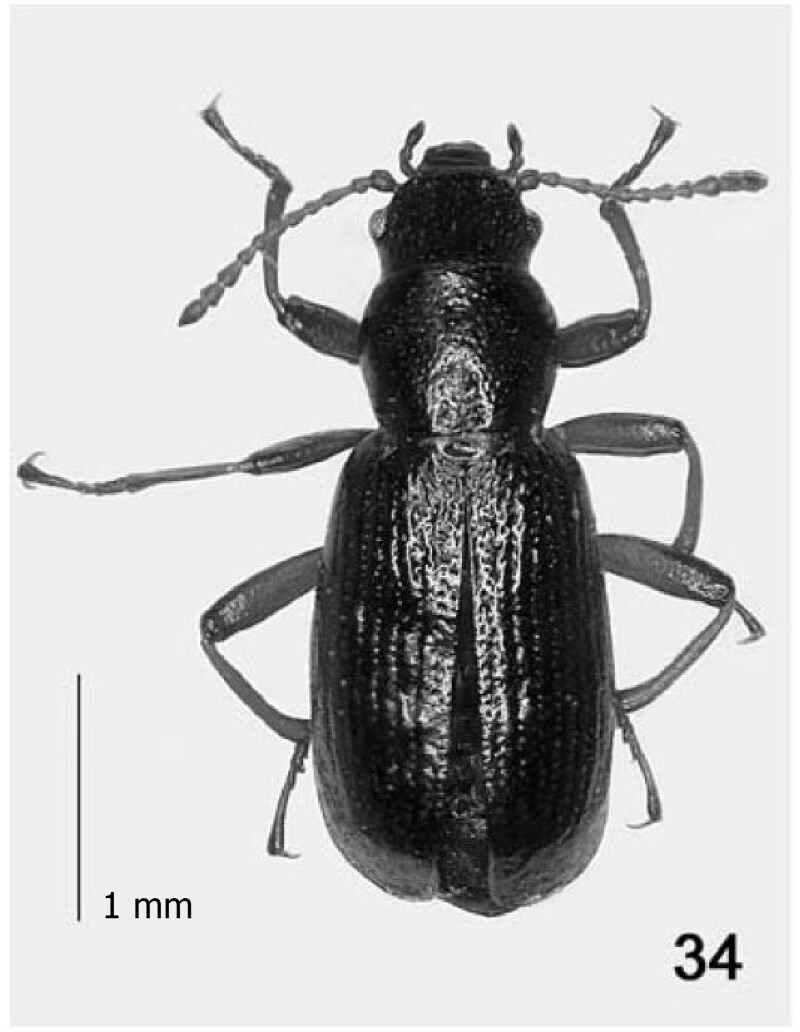
*Aegialites californicus,* neotype (photo taken by Zerche 2004, pp 56) scale: 1 mm.

To increase the number of genetic markers available, we here present the complete mitochondrial genome of *A. californicus* (Motchoulsky, 1845) from Sonoma County, California, assembled with genome skimming sequencing data. The mitochondrial genome can serve as a reference for further molecular work and help the research on taxonomic placement and systematics of the genus (Figure 1).

## Material and methods

A living adult specimen of *A. californicus* was collected and directly placed in 100% ethanol. The specimen label data are as follows: U.S.A., California, Sonoma Co., 1.4 km SSE Carmet, 38°21.709’N 123°04.163’W WGS84, *h* = 0 m, accuracy 4 m, extent 2 m, splitting (crevices in) coastal/tidal rocks (5 m wide boulder) with chisel, V.I.Gusarov & M.N.Haugen leg., 23.xi.2019. The specimen was collected legally, as no special permits for collection or export was necessary as informed by the California Department of Fish and Wildlife. A voucher specimen was deposited in the insect collection at the Natural History Museum of the University in Oslo (https://www.nhm.uio.no/english/collections/zoological/insect/, Vladimir Gusarov, Vladimir.gusarov@nhm.uio.no) with specimen accession number ZMUN 10087480. The species identification was carried out by Vladimir Gusarov based on morphological examinations following the descriptions of Zerche (2004). *A. californicus*, is morphologically most similar to the species *A. farallonensis* and *A. rfuchsii*, but is differentiated from them by being more metallically iridescent, having a more slender antenna and wide open anterior coxae toward the rear. Additionally, the pronotum of *A. californicus* is widest shortly just before the center, it is hardly narrower posteriorly than anteriorly, in *A. farallonensis* it is widest well before the center, much more narrowed posteriorly than anteriorly narrowed. The punctuation of the pronotum is quite fine in *A. californicus* and not stronger than the punctuation of the head; the interspaces on the pronotum measure about two to three times the diameter. In *A. farallonensis* the punctuation of the pronotum is stronger and much denser than in *A. californicus*, the punctuated interspaces measure about half the diameter, the punctuation is stronger than the punctuation of the head. In *A. fuchsii* the punctuation of the pronotum is somewhat stronger, the interspaces measure in places only a third of the diameter and are wrinkled.

The abdomen was dissected from the body with a sterile needle and tweezers, transferred to a new 1,5 mL Eppendorf tube with 100% ethanol before it was shipped on ice to StarSEQ, Mainz, Germany where genomic DNA extraction, library prep and sequencing was performed. A NEBNext® Ultra™ II FS DNA Library Prep kit (New England Biolabs) was utilized, and sequencing was done on the Illumina platform NextSeq 500 with paired end reads and 2 × 150 bp insert size.

Raw data were assembled using metaSPAdes (Nurk et al. 2017) and the linear contig produced by metaSPAdes was manually curated in Mega 7 (Kumar et al. 2016) and the 55 bp identical sequence overlap found in the area believed to be the control region was deleted to ensure the circularization of the mitochondrial genome. The mitochondrial sequence was then annotated using the MITOS2 web server (Donath et al. 2019). The annotation was manually edited so that the first gene feature of the sequence file was *cox1*. The read coverage depth of the Illumina reads was calculated using Bowtie2 (Langmead and Salzberg 2012) and SAMtools (Danecek et al. 2021), and the resulting coverage map was created using Matplotlib (Hunter 2007) (Supplementary Figure 1). The complete mitochondrial sequence of *A. californicus* was deposited at the National Center for Biotechnology Information GenBank under accession number OR168935. The circular mitochondrial genome map was generated using Chloroplot (Zheng et al. 2020).

To validate the phylogenetic position of *A. californicus*, its 13 protein coding genes, and those of 13 other species of Coleoptera were used to create a maximum-likelihood phylogenetic tree using IQ-TREE version 2.2.0 (Minh et al. 2020). Each gene from all 14 species was individually aligned in Mega 7 (Kumar et al. 2016) with the MUSCLE algorithm for codons (Edgar 2004), and a concatenated dataset of all 13 genes was created using FASconCAT-G_v1.05 (Kück and Longo 2014). The phylogenetic inference was run with 1000 bootstrap replicates and the option MFP + MERGE implementing the ModelFinder greedy partitioning strategy (Cameron 2014). The 13 sequences was selected non-randomly using NCBI blastn with *cox1* of *A. californicus* as query with the following settings: taxID limited to superfamily Tenebrionoidea (TaxID 71527), sequence length 12,000-25,000, sequence similarity threshold 80-100%. From the resulting list at NCBI, 11 complete mitochondrial genomes was selected for download and two partial ones. Of the 13 mitochondrial genomes downloaded from NCBI, three other sequences come from the family Salpingidae (*Salpingus planirostris, Lissodema cursor* and *Vincenzellus ruficollis*).

## Results

**Figure 2.**
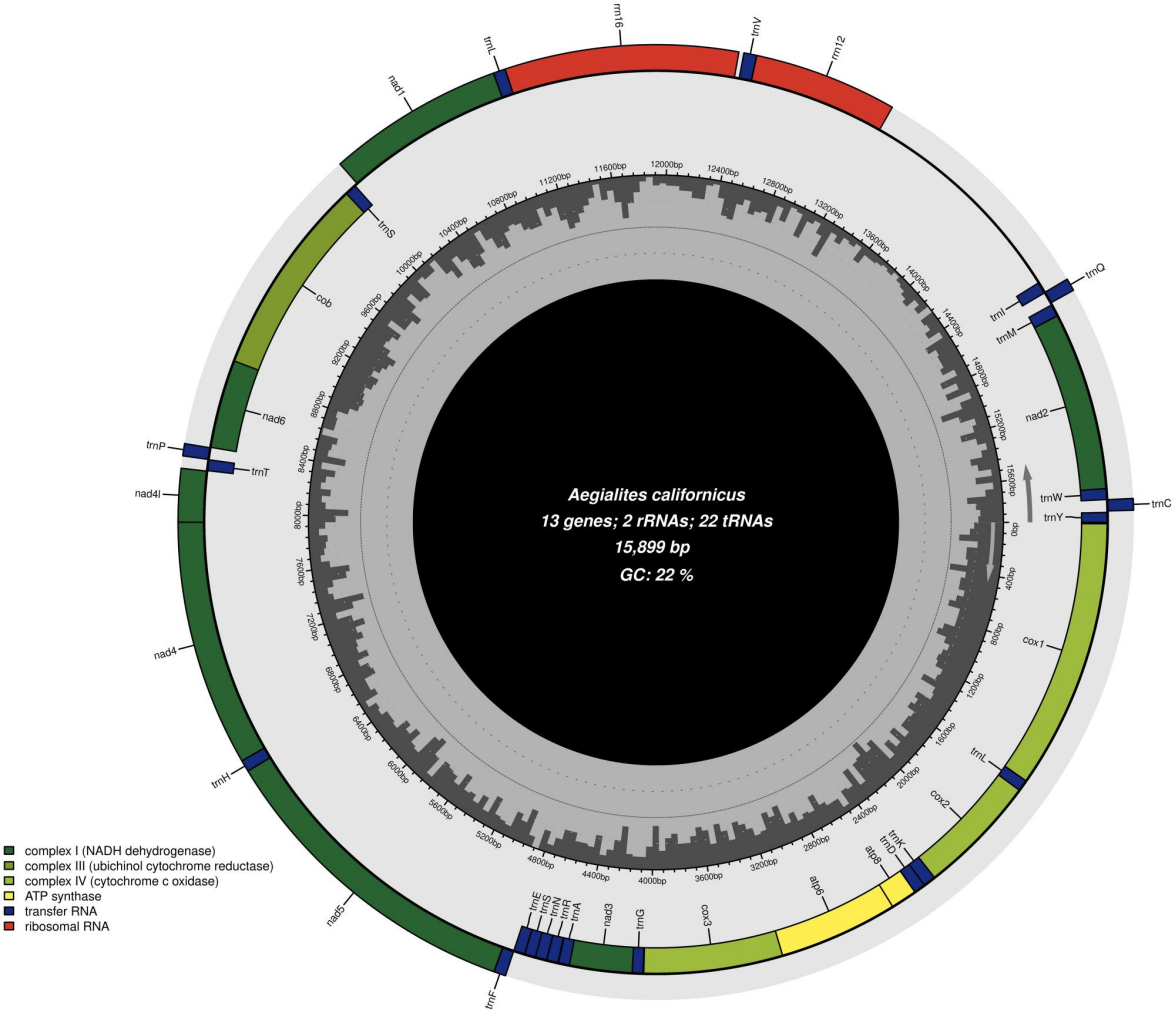
Mitochondrial genome map of *A. californicus* generated by Chloroplot (Zheng et al. 2020*).* Overlapping features are excluded for a better graphical representation of the architecture of the genome. The dark grey areas inside the map represents GC-content, genes on the outside of the map are transcribed counterclockwise, and genes on the inside are transcribed clockwise as informed by the arrows in the figure.

The mitochondrial genome of *A. californicus* is 15,899 bp and consists of 13 protein-coding genes (PCG), two ribosomal RNA (rRNA) genes, and 22 transfer RNA (tRNA) genes. The mitochondrial genome has an AT-bias, with an AT-content of 78% (*A* = 39%, *T* = 39%, *C* = 13%, *G* = 9%). The non-coding control region (also known as D-loop or origin of replication) could possibly be between *trnI* and *rrnS* (Figure 2), but the annotation by MITOS2 was not certain in this respect, and hence not visualized in the genome plot. The gene order of the 13 PCGs is visualized in the genome plot in Figure 2. All the 13 PCGs have a typical ATN (Met) start codon; five genes (*atp6*, *cox3*, *nad4*, *nad4l* and *cob*) have ATG and eight genes (*atp8*, *cox1, cox2, nad1, nad2, nad3, nad5* and *nad6*) have ATA. All the PCGs have a typical TAN stop codon; six genes (*atp6*, *cox3*, *nad2*, *nad4l, nad6* and *cob*) have TAA and three genes (*atp8*, *nad1* and *nad3*) have TAG. Two genes (*cox1* and *cox2*) have TGA and two genes (*nad4* and *nad5*) are found to have TGG, so in these four genes, TAA stop codon is completed by the addition of 3′ A residues to the mRNA.

The 22 tRNA genes in *A. californicus* are interspersed throughout the coding region and range from 63 bp (*trnT, trnW*, *trnS* and *trnH*) to 71 bp (*trnK*). An overlap of seven bp was detected between the protein coding genes *atp6* and *atp8* and one bp overlap between *atp6* and *cox3* and between *nad6* and *cob*. Additional overlaps between t-RNA genes and PCGs were discovered; the longest being 42 bp between *cox2* and *trnL*. The ribosomal RNA genes, *rrnL* (also known as *l-rRNA* or *16S rRNA*) and *rrnS* (*s-rRNA*, *12S*) were 1278 and 772 bp long, respectively.

Based on ModelFinder in IQtree and the Bayesian information criterion (BIC) the best fitting partition scheme and the corresponding substitution models comprised three partitions with the following genes grouped together: *atp6*, *cob*, *cox1*, *cox2*, *cox3*: mtART + I+R3; *atp8*, *nad2*, *nad3*, *nad6*: mtART + F+R4; *nad1*, *nad4*, *nad4l*, *nad5*: mtART + F+R4.

**Figure 3.**
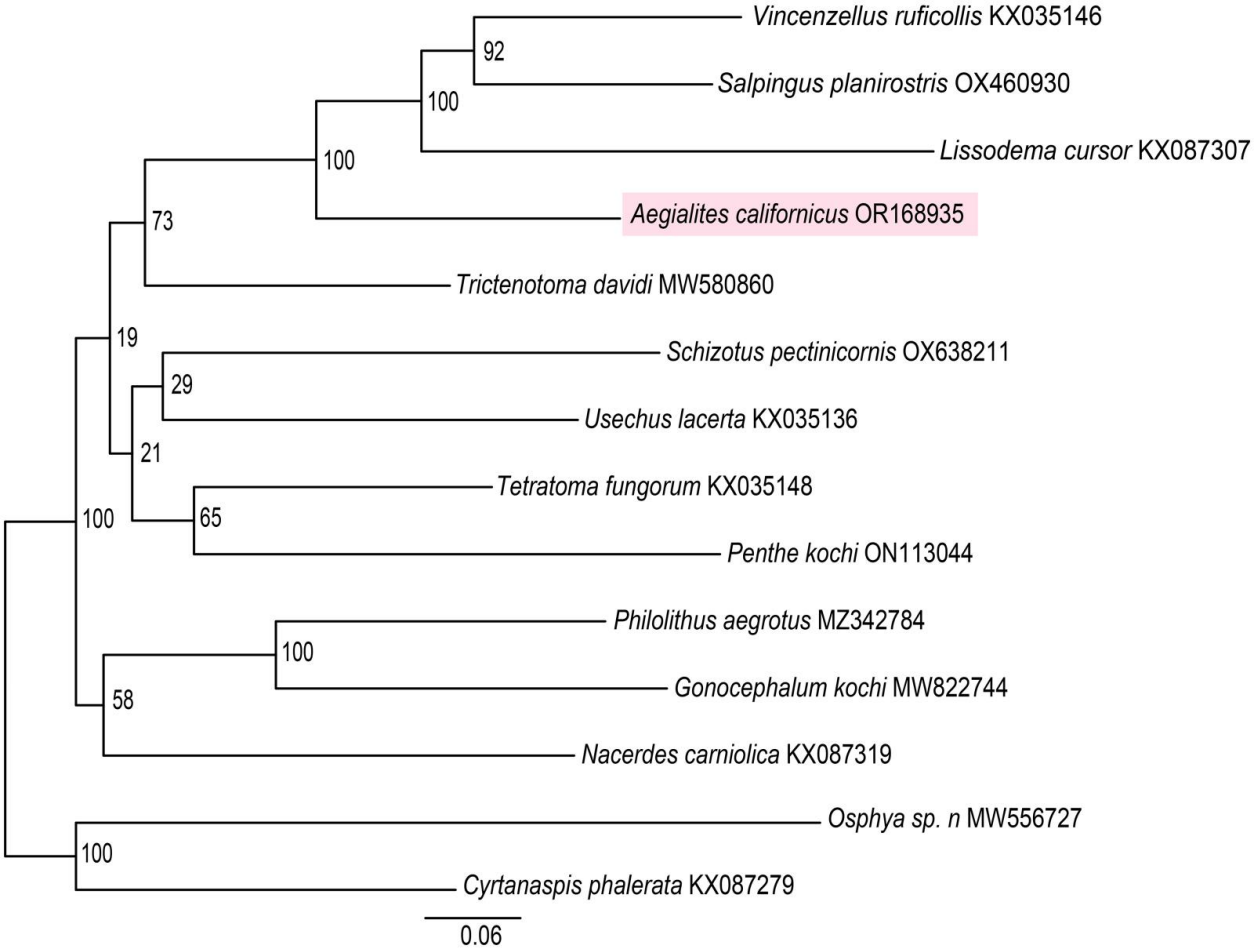
Maximum-likelihood tree for *Aegialites californicus* (highlighted) and 13 additional insect taxa from superfamily Tenebrionoidea derived from the following records: *Vincenzellus ruficollis* KX035146 (unpublished), *Salpingus planirostris* OX460930 (unpublished), *Lissodema cursor* KX087307 (unpublished), *trictenotoma davidi* MW580860 (Sheng et al. 2021), *schizotus pectinicornis* OX638211 (unpublished), *usechus lacerta* KX035136 (unpublished), *tetratoma fungorum* KX035148 (unpublished), *penthe kochi* ON113044 (unpublished), *philolithus aegrotus* MZ342784 (Smith et al. 2021), *gonocephalum kochi* MW822744 (unpublished), *nacerdes carniolica* KX087319 (unpublished), *osphya sp. n*. MW556727 (Liu et al. 2023) and *cyrtanaspis phalerata* KX087279 (unpublished). The GenBank accession numbers for the sequences are indicated next to the species names. Bootstrap values are found close to the nodes and a scale bar indicating the mean number of nucleotide substitutions per site is located at the bottom.

The resulting tree based on 13 mitochondrial genes places *A. californicus* as sister to the clade composed of *V. ruficollis*, *S. planirostris* and *L. cursor,* the three other representatives of family Salpingidae, with a bootstrap support of 100 (Figure 3). The four members of Salpingidae are monophyletic in this dataset and they are found closest to *T. davidi* from family Trictenotomidae.

## Discussion and conclusions

In this study we assembled and described the characteristics of the mitochondrial genome of *A. californicus*. The mitochondrial genome is found to be 15,899 bp and consists of 13 protein-coding genes (PCG), two ribosomal RNA (rRNA) genes, and 22 transfer RNA (tRNA) genes, with gene order found to be identical as in the ancestral insect genome (Cameron 2014). The phylogentic analysis confirms the placement of *A. californicus* as sister to three representatives of family Salpingidae. The four salpingids are found to be monophyltic and found closest to *T. davidi* as expected according to Hu et al. (2020). Lastly, this study provides genetic data for further analyses and studies of genus *Aegialites*, family Salpingidae and superfamily Tenebrionoidea.

## Supplementary Material

Supplemental Material

Supplemental Material

## Data Availability

The data that support the findings of this study are openly available in NCBI GenBank database at https://www.ncbi.nlm.nih.gov with the accession number OR168935, which permits unrestricted use, distribution, and reproduction in any medium, provided the original work is properly cited. Associated BioProject, SRA, and BioSample accession numbers are PRJNA985217, SRR25009919 and SAMN35791155, respectively.
