# Supplementary material for: The complete mitochondrial genome of Aegialites californicus (Motchoulsky, 1845) (insecta: coleoptera: salpingidae)
Source: Mitochondrial DNA B Resour. 2024 Jan 30;9(1):214–8. doi: 10.1080/23802359.2024.2309255 (PMC10829811; doi:10.1080/23802359.2024.2309255)
Supplement: Supplemental Material [file TMDN_A_2309255_SM2214.docx]

Biogeographical note

**Marianne Nilsen Haugen**

https://www.nhm.uio.no/english/about/organization/research-collections/people/marinhau/index.html

Currently a PhD-student with the Frontiers in Evolutionary Zoology (FEZ) group at the Natural History Musum of the University in Oslo, working with beetle genus Aegialites. She studies the taxonomy of the genus as well as the capacity for dispersal and hybridization using modern molecular techniques.

**Vladimir I. Gusarov**

<https://www.nhm.uio.no/english/about/organization/research-collections/people/vladig/index.html>

Vladimir I. Gusarov is an Associate Professor and curator of the insect collection at the Natural History Museum, University of Oslo, Norway. He has a Ph.D. in Entomology (1992), St. Petersburg State University, Russia. The area of taxonomic expertise is rove beetles (family Staphylinidae). Research interests are mainly in the field of insect systematics and phylogenetics. Current projects include research on several groups of beetles, Norwegian inventory of Laboulbeniales fungi parasitizing beetles of the superfamily Staphylinoidea, and student training.

**Torsten H. Struck**

https://www.nhm.uio.no/english/about/organization/research-collections/people/torsths/index.html

He works at the Natural History of Museum of the University of Oslo as a "Full Professor of Evolutionary Genomics and Curator of the Helminth collection" since 2015. In his research,he is interested in invertebrate diversity at all taxonomic levels; this includes phylogeny as well as cryptic species and stasis, especially to what extent genomic limitations or possibilities influence evolvability. His research is predominantly on marine invertebrates, but recently also insects. Methodically, he employ a comparative approach using molecular and morphological data.
