# Supplementary figures and images for: The complete mitochondrial genome of Aegialites californicus (Motchoulsky, 1845) (insecta: coleoptera: salpingidae)
Source: Mitochondrial DNA B Resour. 2024 Jan 30;9(1):214–8. doi: 10.1080/23802359.2024.2309255 (PMC10829811; doi:10.1080/23802359.2024.2309255)

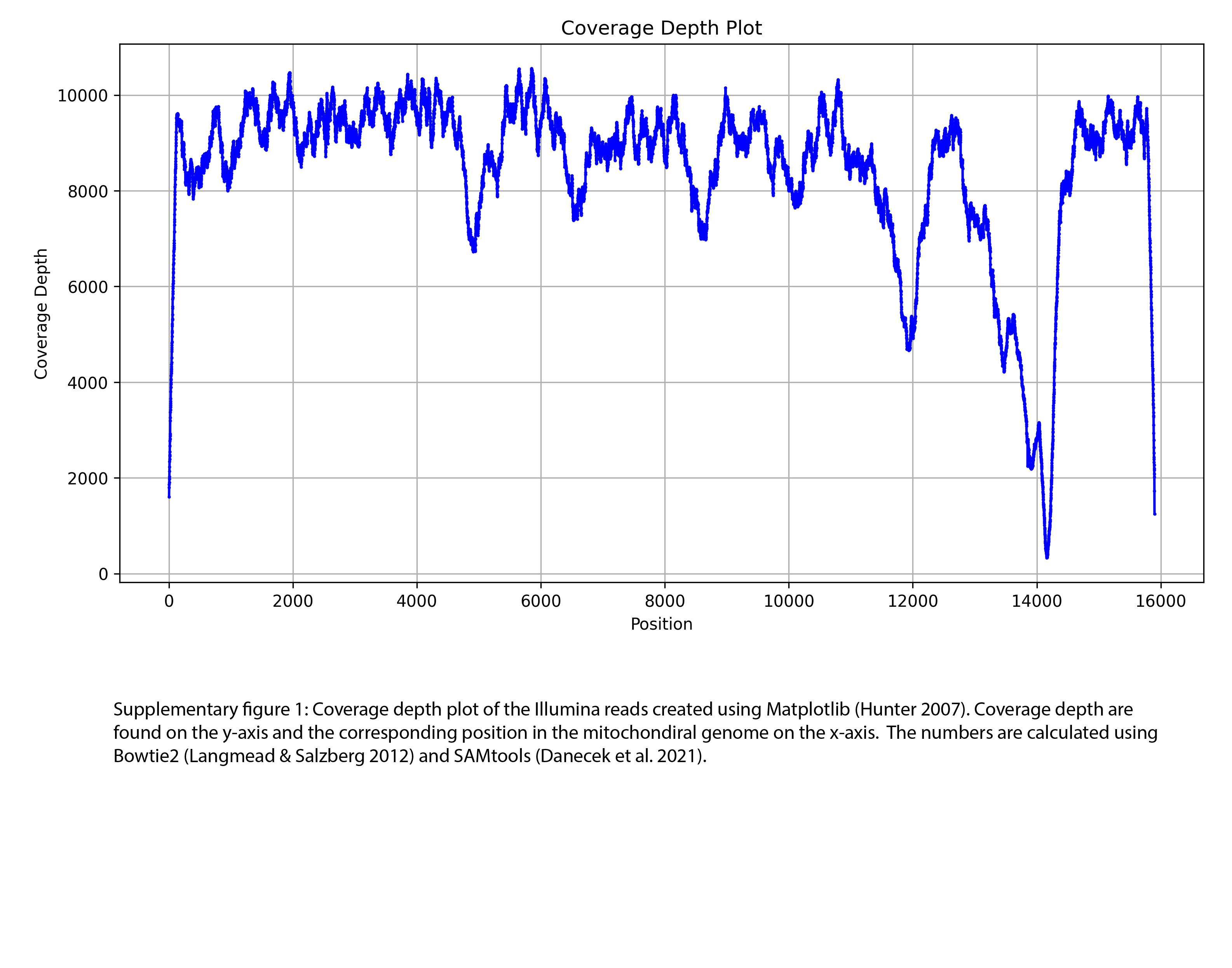

Supplement: Supplemental Material [file TMDN_A_2309255_SM2210.jpg]
